# Reply to: The Use of the Newest Vital Sign in Children

**DOI:** 10.3928/24748307-20190122-04

**Published:** 2019-02-05

**Authors:** Carol J. Howe, Gina Alexander, Christine Van Scoyoc, Jada L. Stevenson

**Affiliations:** Fort Worth, TX; Fort Worth, TX; Dallas, TX; Fort Worth, TX

## Response:

We thank Dr. Barry Weiss for his comments about our study on the use of the NVS in children. We certainly have consensus that the NVS is not an appropriate instrument to measure children's health literacy. However, we assert that it is vital for clinicians and researchers alike to assess the health literacy of all patients, including children—particularly those who are school-aged and making decisions about their nutrition independently of their parents. Adoption of healthy or unhealthy behaviors begins in childhood and continues into adulthood. Given the high rate of overweight and obesity among children in the United States, the importance of child health literacy cannot be overstated.

We decided to use the NVS in an exploratory pilot study at a local children's museum because Driessnack, Chung, Perkhounkova, and Hein (2014) reported the NVS to be a feasible tool for children age 7 to 13 years. Most of the children in our study scored zero, so we had insufficient variability to test associations as we originally had planned. We described educational standards that support the assertion that the NVS may be testing children on something that they would not be expected to know. We could have chosen not to report this data, but instead we decided to report on what we learned about the NVS in a sample of children, anticipating that our findings would add to the important discussion of health literacy and children.

We agree with Dr. Weiss that health literacy researchers should take a holistic approach that involves patients, health care providers, and health care systems to improve health literacy. However, parent health literacy is an insufficient proxy for children's health literacy. We need to expand our health literacy research efforts to include children intentionally. For example, we need to understand the process by which children develop health literacy and we must identify best practices to build their health literacy skills. For this effort, we need reliable and valid health literacy measures for children. Many of the instruments we use in pediatric research have been adapted from adult tools. In our article, we discussed ways that the NVS could possibly be modified for use in children. We welcome a candid discussion on child health literacy and thank *HLRP: Health Literacy Research and Practice* for providing a forum for health literacy researchers and clinicians.

*Carol J. Howe, PhD, RN, CDE* Fort Worth, TX*Gina Alexander, PhD, MPH, MSN, RN* Fort Worth, TX*Christine Van Scoyoc, MEd* Dallas, TX*Jada L. Stevenson, PhD, RDN, LD* Fort Worth, TX
